# Allyl Isothiocyanate Increases MRP1 Function and Expression in a Human Bronchial Epithelial Cell Line

**DOI:** 10.1155/2014/547379

**Published:** 2014-01-14

**Authors:** Dian-lei Wang, Chen-yin Wang, Yin Cao, Xian Zhang, Xiu-hua Tao, Li-li Yang, Jin-pei Chen, Shan-shan Wang, Ze-geng Li

**Affiliations:** ^1^Anhui University of Chinese Medicine, Hefei, Anhui 230038, China; ^2^The Fourth People's Hospital of Hefei, Hefei, Anhui 230022, China; ^3^The First Hospital Affiliated to Anhui University of Chinese Medicine, Hefei, Anhui 230031, China

## Abstract

Multidrug resistance-associated protein 1 (MRP1), a member of the ATP-binding cassette (ABC) superfamily of transporters, plays an important role in normal lung physiology by protecting cells against oxidative stress and toxic xenobiotics. The present study investigates the effects of allyl isothiocyanate (AITC) on *MRP1* mRNA and MRP1 protein expression and transporter activity in the immortalised human bronchial epithelial cell line 16HBE14o-. *MRP1* mRNA and MRP1 protein expression in 16HBE14o- cells that were treated with allyl isothiocyanate were analysed by real-time PCR assay and Western blotting. The transport of carboxyfluorescein, a known MRP1 substrate, was measured by functional flow cytometry to evaluate MRP1 activity. Treatment with AITC at concentrations of 5–40 **μ**M increased MRP1 protein levels in a concentration-dependent manner. AITC treatments at concentrations of 1–40 **μ**M caused concentration-dependent increases in *MRP1* mRNA levels that were up to seven times greater than the levels found in control cells. Finally, AITC treatment at concentrations of 5–40 **μ**M significantly increased MRP1-dependent efflux in 16HBE14o- cells. These results suggest that AITC can increase the expression and activity of MRP1 in 16HBE14o- cells in a concentration-dependent manner. The upregulation of MRP1 activity and expression by AITC could produce therapeutic effects in the treatment of lung disease.

## 1. Introduction

Isothiocyanates (–N=C=S) are naturally occurring compounds that are primarily synthesised and stored in plants and cruciferous vegetables, including Brussels sprouts, broccoli, cabbage, kale, and radishes, as glucosinolates. Isothiocyanates have shown significant cancer chemopreventive activity [1]. Allyl isothiocyanate (AITC), a hydrolysis product of the glucosinolate sinigrin, has significant antimicrobial activity and possesses potential anticancer activity against cancers such as colorectal and bladder cancers [2–6]. AITC causes cell cycle arrest and apoptosis in cancer cell lines derived from different tissues and modulates the levels of many genes and proteins that are known to be involved in cancer cell survival and proliferation. AITC-induced stomatal closure is partially inhibited by an NADPH oxidase inhibitor and is completely inhibited by glutathione monoethyl ester [7]. The oral bioavailability of AITC is extremely high, at nearly 90% [8]. Isothiocyanates such as sulforaphane (SF) and erucin (ER) are known to enhance the expression levels of Phase II detoxification enzymes. Isothiocyanate also increases the levels of multidrug resistance protein 1 (MRP1) and multidrug resistance protein 2 (MRP2) in human carcinoma cell lines [9].

MRP1 (190 kDa), a member of a subfamily of the ATP-binding cassette (ABC) superfamily of transport proteins, was first detected in small cell lung cancer [10]. MRP1 is highly expressed in normal human lung tissue [11], particularly on the basolateral side of human bronchial epithelial cells [12, 13]. Glutathione, glucuronate, and sulphate-conjugated organic anions are MRP1 substrates [14, 15]. Endogenous leukotriene C_4_ (LTC_4_) and glutathione disulphide (GSSG) are transported by MRP1 [16, 17]. Thus, MRP1 could play an important role in normal lung physiology by protecting cells against oxidative stress and toxic xenobiotics [18, 19].

The purpose of the present study was to investigate whether AITC changed MRP1 expression and activity *in vitro* in the human bronchial epithelial cell line 16HBE14o-.

## 2. Materials and Methods

### 2.1. Materials

AITC was purchased from Anhui Haibei Import and Export Company (Hefei, Anhui, China). RPMI 1640 medium was obtained from Gibco. N-Acetylcysteine (NAC), 5-carboxyfluorescein diacetate (5-CFDA), and MK571 were purchased from Sigma-Aldrich. A monoclonal anti-MRP1 antibody (MRPr1) was purchased from Alexis Biochemicals (San Diego, CA, USA). Trizol reagent was purchased from Invitrogen (CA, USA). Dimethyl sulphoxide (DMSO) was purchased from Rui-Bio Company. All other chemicals were commercially available and of reagent grade.

The human bronchial epithelial cell line 16HBE14o- was purchased from Shanghai Fuxiang Biological Technology Company. Cells were grown in RPMI 1640 supplemented with 10% foetal bovine serum, 2 mM L-glutamine, penicillin (10 units/mL), and streptomycin (10 g/mL). Cells were maintained in 5% CO_2_ at 37°C in a humidified chamber. Cells were routinely subcultured prior to reaching 80% confluence. AITC was dissolved in DMSO.

### 2.2. Cell Viability Assay

16HBE14o- cells were seeded into 96-well dishes at a density of 1 × 10^4^ cells/well. After 24 h, the medium was removed and replaced with medium containing 0–80 *μ*M AITC for 24 h and 48 h. After incubation with AITC, the cells were incubated in 20 *μ*L of a 5 mg/mL solution of MTT at 37°C for 4 h and then lysed in 150 *μ*L DMSO for 10 min at room temperature with agitation. The absorbance of each well was measured at 490 nm in a microplate reader. The absorbance at 490 nm is proportional to the number of metabolically active/living cells in the culture.

### 2.3. Real-Time Polymerase Chain Reaction (PCR)

Additional 16HBE14o- cells were plated in six-well dishes at a density of 1 × 10^5^ cells/well. 24 h later, the medium was removed and replaced with a treatment medium containing either 0–40 *μ*M AITC or NAC (the positive control drug) (8 × 10^−4 ^M). Cell total RNA was extracted from the 16HBE14o- cells using Trizol reagent according to the manufacturer's instructions. The concentrations and A260/A280 ratios of the isolated RNAs were determined from the absorbance at 260 and 280 nm in a Hitachi spectrophotometer (model U1100) and the integrity was verified by agarose gel electrophoresis. cDNA was generated using the High-Capacity cDNA Archive Kit according to the manufacturer's instructions. Real-time PCR was performed using the SYBR Green MasterMix system (Applied Biosystems) according to the manufacturer's instructions on an ABI 7500 real-time PCR machine (Applied Biosystems). Following the reverse transcription reaction, 2 *μ*L of the resultant cDNA was used for PCR amplification reaction as follows: 10 min at 95°C. followed by 40 cycles of 15 s at 95°C and 1 min at 60°C The primers used were MRP1 forward 5′-CCTGGAGCTGGCCCACCTGA-3′ and reverse 5′-CGCTGCCCGACACTGAGGTT-3′. GAPDH was used as housekeeping gene, forward 5′-CAAGGCTGTGGGCAAGGT-3′ and reverse 5′-GGAAGGCCATGCCAGTGA-3′.

### 2.4. Western Blotting Analysis

Additional 16HBE14o- cells were plated in six-well plates at a density of 1 × 10^6^cells/well. 24 h later, the medium was removed and replaced with a treatment medium containing either 0–40 *μ*M AITC or NAC (8 × 10^−4 ^M). The protein concentrations were measured by using the BioRad Protein Assay Reagent according to the manufacturer's instructions. Total protein was separated by electrophoresis on a 10% SDS-polyacrylamide gel and electroblotting on PVDF (polyvinylidene fluoride) membranes. Membranes were blocked overnight at 4°C in a buffer containing 5% nonfat milk, 1% Tween-20, 1 M Tris-HCl, and 0.5 M NaCl and for 2 h at room temperature with agitation. Membranes were probed with a monoclonal antibody against MRP1 for 2 h at room temperature with agitation, washed 3 times in wash buffer (20 mM Tris base, 137 mM NaCl, and 1% Tween 20, pH 7.6), and incubated with a polyclonal horseradish-peroxidase-conjugated secondary antibody for 2 h. After washing, membranes were developed using the ECL Plus system according to the manufacturer's instructions. Kodak 1D image analysis software was used to analyse the Western blotting results.

### 2.5. Flow Cytometry

To determine MRP1-mediated transport, 16HBE140o- cells were incubated with a medium containing 1 *μ*M 5-CFDA for 1 h. The compound 5-CFDA is a nonpolar, nonfluorescent compound that diffuses freely into cells, where it is converted to carboxyfluorescein (CF), which is a substrate for efflux by MRP1. To establish the effect of AITC on MRP1-mediated activity, 1 × 10^6^ cells were incubated in 0.5 mL RPMI 1640 medium with or without the addition of AITC for 24 h. Cells were washed twice with cold phosphate-buffered saline (PBS) and incubated for 60 min at 37°C in medium with or without 20 *μ*M MK-571, which was used as a specific inhibitor of MRP1 activity. In addition NAC was as the positive control drug to induce MRP1 activity. After incubation, cells were detached and centrifuged at 500 ×g for 5 min, and the pellets were suspended in 1 mL of ice-cold PBS and immediately placed on ice. The fluorescence of the CF retained within the cells was analysed by flow cytometry in 30 min using an ABI LSRII flow cytometer (Applied Biosystems). We measured 10,000 events per sample (living cells). Samples were excited at 488 nm using an argon laser, and the emission fluorescence was detected at 530 nm. The Winlist 5.1 program (Verity Software House Inc., Topsham, ME, USA) was used to calculate the mean fluorescence intensity (MFI) values.

### 2.6. Statistical Analysis

Paired Student's *t*-tests or independent sample *t*-tests were used to calculate significant differences. Differences were considered significant when *P* < 0.05. Statistical analyses were performed with SPSS 17.0 (SPSS Inc., Chicago, IL).

## 3. Results

**Figure 1:**
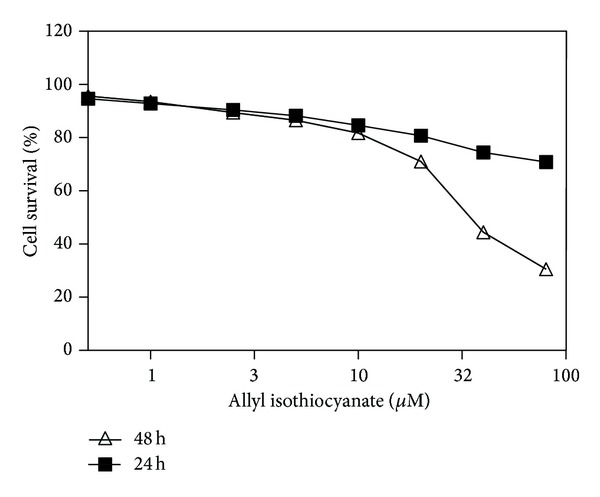
Survival of 16HBE14o- cells in the presence of AITC, as determined by the MTT assay. Cells were incubated with 0–80 *μ*M AITC for 24 and 48 h. The IC_50_ concentration was 35.86 ± 0.037 *μ*M with 0–80 *μ*M AITC for 48 h (mean ± SE, *n* = 4).

The cytotoxicity of AITC in a 16HBE14o- cell line was evaluated using the MTT assay, which ensured that the cell number and cell proliferation were optimized for subsequent experiments. After incubating for 24 h, the data showed that AITC had no significant effect on human bronchial epithelial cell line 16HBE14o- at concentration from 1 to 40 *μ*M, and at 80 *μ*M AITC significantly decreased cell viability in 16HBE14o- cells (Figure 1). The IC_50_ for AITC in 16HBE14o- cells was 35.86 ± 0.037 *μ*M when 16HBE14o- was incubated for 48 h. Therefore, AITC concentrations in the range of 1–40 *μ*M and treatment period of 24 h were selected for subsequent experiments.

**Figure 2:**
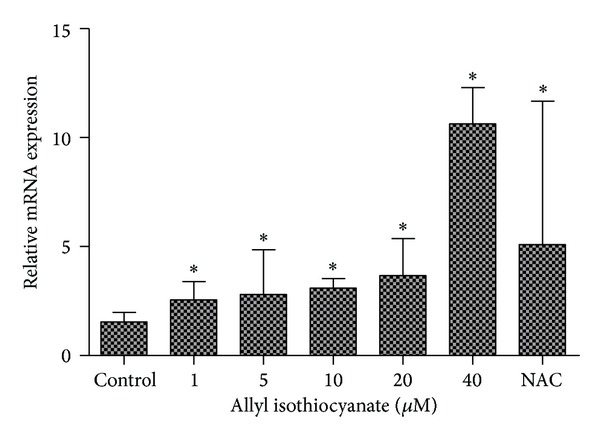
The effect of AITC or NAC (8 × 10^−4 ^M) treatment on *MRP1* mRNA expression in 16HBE14o- cells. Cells were exposed to treatments for 24 h. Data represent means ± SE of two independent experiments, performed in triplicate. *P < 0.05.

The levels of *MRP1* mRNA in 16HBE14o- cells after AITC treatments were determined by real-time reverse-transcription PCR (Figure 2). AITC significantly increased *MRP1* mRNA levels at concentrations ≥1 *μ*M. The increase in *MRP1* mRNA levels induced by AITC treatment was dependent on the concentration of AITC, with up to a sevenfold increase over levels found in untreated control cells. These results demonstrate that *MRP1* mRNA expression could be effectively upregulated by AITC in 16HBE14o- cells. In the 16HBE14o- cells, treatment with NAC resulted in similar changes in *MRP1* mRNA levels to those obtained after treatment with AITC.

**Figure 3:**
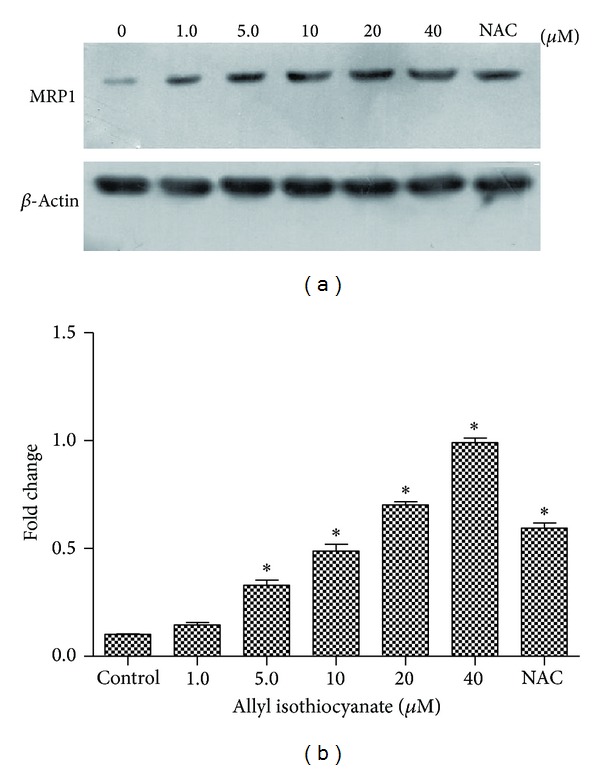
(a) The effect of AITC or NAC (8 × 10^−4 ^M) treatment on MRP1 protein levels. Cells were treated for 24 h. Equal protein loading was confirmed by the detection of *β*-actin. (b) Densitometric analysis of data from 16HBE14o- cells showing the effects of AITC on MRP1 levels. Data are presented as the mean ± SE (*n* = 3); *P < 0.05.

The effect of AITC on the expression of MRP1 protein was evaluated in 16HBE14o- cells. The complete Western blotting corresponding molecular weight range 175~200 kDa was shown in Figure 3(a). An increase in MRP1 protein levels was not observed in 16HBE14o- cells after treatment with 1.0 *μ*M AITC, but AITC concentrations of 5–40 *μ*M increased MRP1 protein levels (Figure 3(b)). AITC increased MRP1 protein levels in a concentration-dependent manner compared to untreated control cells. The positive control drug NAC also increased MRP1 protein levels in 16HBE14o- cells.

**Figure 4:**
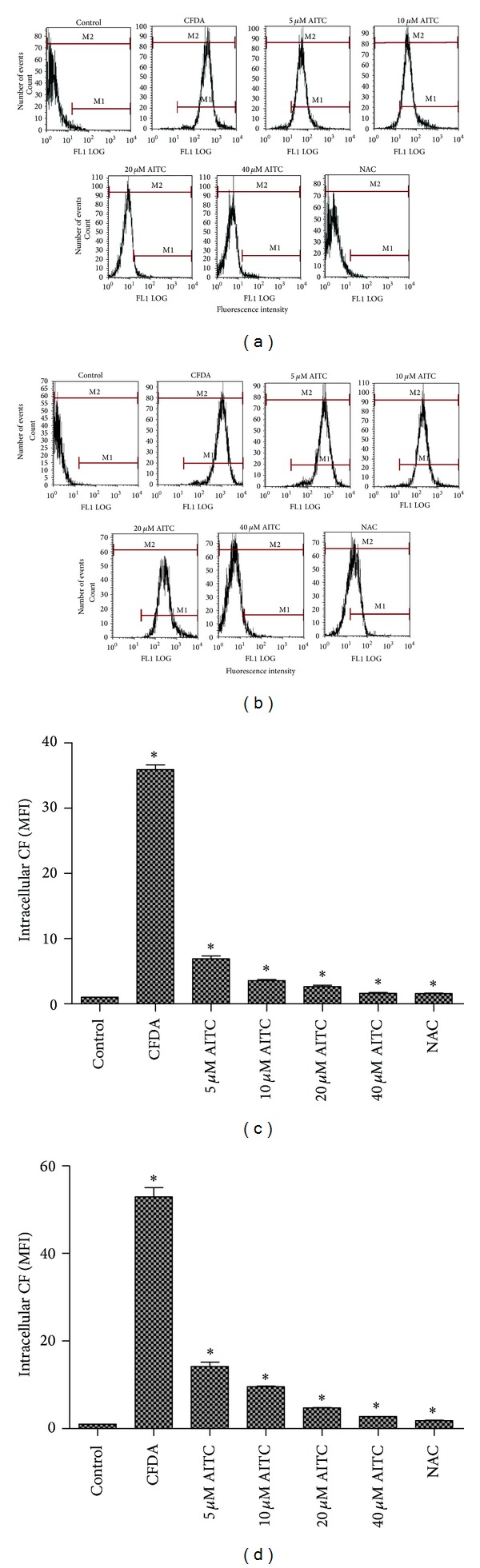
The effect of AITC or NAC treatments on MRP1-dependent efflux in 16HBE14o- cells. (a) Cells were stimulated with ATIC or NAC (8 × 10^−4 ^M) for 24 h. (b) Cells were stimulated with AITC or NAC (8 × 10^−4 ^M) for 24 h and then incubated with MK-571 (MRP1 inhibitor) for 1 h. (c) Intracellular CF (MFI) retention as measure of MRP1 function in 16HBE14o- cells incubated without MK-571. (d) Intracellular CF (MFI) retention as measure of MRP1 function in 16HBE14o- cells incubated with MK-571. Data are presented as the mean ± SE (*n* = 3) and *P < 0.05 AITC or NAC treated versus untreated control. CF, carboxyfluorescein; MFI, mean fluorescence intensity.

To determine whether increased MRP1 protein levels resulted in increased MRP1 activity in 16HBE14o- cells, the cells were loaded with 5-CFDA, and the disappearance of the fluorescent efflux substrate CF was evaluated by flow cell cytometry 30 min after loading. Intracellular CF in 16HBE14o- cells decreased with increasing AITC concentrations (*P* < 0.05) (5–40 *μ*M; Figure 4(a)). Treatment of 16HBE14o- cells with AITC at concentrations of 5–40 *μ*M resulted in significant increases in MRP1-dependent efflux (Figure 4(c)). The intracellular fluorescence intensity was significantly increased after coincubation with the MRP1 inhibitor MK571 compared to the respective controls (*P* < 0.05) (Figure 4(b)). NAC had a similar significant effect on the accumulation of CF at 8 × 10^−4 ^M. This result showed that AITC could increase MRP1 activity.

## 4. Discussion

We have reported for the first time that AITC induces concentration-dependent increases in the levels of *MRP1* mRNA and MRP1 protein expression and functional activity in 16HBE14o- cells. Our findings showed that treating 16HBE14o- cells with AITC increased the expression of *MRP1* mRNA and this increase was associated with an increase in MRP1 protein expression and transporter activity. Our previous study showed that the plasma concentration of AITC at 300 min was more than 1 *μ*g/mL [20] and the concentration of AITC in the lung tissue was 0.65 *μ*g/g at 60 min after intravenous injection of AITC 20 mg·kg^−1^. Compared with the AUC of AITC after intravenous administration, the absolute bioavailability of AITC after i.g. administration was 84.95%, which was consistent with the published data [8]. Compared with the concentration of AITC *in vitro*, the plasma concentration of AITC could be reached in a consistently level *in vivo*.

5-CFDA was used as a model MRP1 substrate and was measured with flow cytometry to evaluate the function of MRP1 [21]. The induction of AITC was observed absolutely from Figures 4(a) and 4(c). In the same time, we used the insufficient MK571 as a positive control for inhibition of MRP1 activity [22], which further confirmed that AITC could increase the function of MRP1 (Figures 4(b) and 4(d)). The results also showed that the intracellular fluorescence intensity was significantly increased after coincubation with the MRP1 inhibitor MK571 compared to the respective controls (Figures 4(a) and 4(c)). Previous reports used flow cytometry to study the function of MRP1 in 16HBE14o- cells and found that NAC increased CF transport by MRP1 in a concentration-dependent manner [22, 23]. The present study showed that NAC increased not only MRP1 functional activity but also the *MRP1* mRNA and MRP1 protein levels.

Chronic obstructive pulmonary disease (COPD) is an inflammatory lung disease [24]; reduced MRP1 expression in the bronchial epithelium of COPD patients compared to healthy subjects has been reported. In a *mrp1*/*mdr1a/1b* triple knockout mouse model, smoke-induced IL-8 production was reduced compared to wild type mice, and this reduction in IL-8 production was associated with a significantly reduced inflammatory response to cigarette smoke [25]. These observations suggest that MRP1 is likely to play an important role in COPD [26, 27]. In traditional Chinese medicine, the seeds of cruciferous herbs, such as *Sinapis albae*, pepperweed (*Lepidium latifolium L.*) and Radish (*Raphanus sativus L.*), are widely used to treat respiratory inflammatory disorders such as asthma and COPD; AITC is the major component in these medicinal plants [28, 29]. We have previously shown that the herbal recipe “Huatan jiangqi,” which has *Sinapis albae* as its major plant component, could alleviate lung inflammation and improve lung function in a rat COPD model; these effects may be due to the regulation of the function and expression of MRP1 in the bronchial epithelium [30]. We speculate that the upregulation of MRP1 activity and expression is a possible explanation for the therapeutic effects of herbal medicines in lung diseases such as COPD. Further study is required to determine whether the clinical effects of these herbal medicines are at least partly due to AITC.

Based on studies in embryonic fibroblast cells from wild type and knockout mice, NFE2-related factor-2 (Nrf2) is important for the expression of constitutive and inducible levels of MRP1 [31–35]. Nrf2 is involved in the cellular response to oxidative stress, stimulating the induction of genes that are regulated by antioxidant response elements (AREs). The Nrf2 pathway has been demonstrated to regulate ARE-dependent pathways in macrophages and monocytes. Furthermore, Nrf2 is an antioxidant-activated transcription factor that recently emerged as a critical regulator of the cellular defence system against oxidative and inflammatory lesions [36]. These studies suggest that the Nrf2 pathway maybe involved in the increases in MRP1 expression that were observed in the present study. More studies are necessary to evaluate the role of Nrf2 and putative AREs in the induction of MRP1 by AITC.

In conclusion, the present study showed that AITC increased the expression and activity of MRP1, a transporter that protects against oxidative stress and toxic xenobiotics in lung tissue, in a concentration-dependent manner. The upregulation of MRP1 activity and expression may be associated with therapeutic benefits in the treatment of lung disease.

## Abbreviations

AITC: Allyl isothiocyanate
MRP1: Multidrug resistance-associated protein 1
ARE: Antioxidant response elements
RT-PCR: Real-time polymerase chain reaction.

## References

[1] Conaway CC, Yang Y-M, Chung F-L (2002). Isothiocyanates as cancer chemopreventive agents: their biological activities and metabolism in rodents and humans. *Current Drug Metabolism*.

[2] Liu T-T, Yang T-S (2010). Stability and antimicrobial activity of allyl isothiocyanate during long-term storage in an oil-in-water emulsion. *Journal of Food Science*.

[3] Wang N, Shen L, Qiu S (2010). Analysis of the isothiocyanates present in three Chinese Brassica vegetable seeds and their potential anticancer bioactivities. *European Food Research and Technology*.

[4] Lau W-S, Chen T, Wong Y-S (2010). Allyl isothiocyanate induces G2/M arrest in human colorectal adenocarcinoma SW620 cells through down-regulation of Cdc25B and Cdc25C. *Molecular Medicine Reports*.

[5] Bhattacharya A, Li Y, Wade KL, Paonessa JD, Fahey JW, Zhang Y (2010). Allyl isothiocyanate-rich mustard seed powder inhibits bladder cancer growth and muscle invasion. *Carcinogenesis*.

[6] Bhattacharya A, Tang L, Li Y (2010). Inhibition of bladder cancer development by allyl isothiocyanate. *Carcinogenesis*.

[7] Khokon MAR, Jahan MS, Rahman T (2011). Allyl isothiocyanate (AITC) induces stomatal closure in Arabidopsis. *Plant, Cell and Environment*.

[8] Bollard M, Stribbling S, Mitchell S, Caldwell J (1997). The disposition of allyl isothiocyanate in the rat and mouse. *Food and Chemical Toxicology*.

[9] Harris KE, Jeffery EH (2008). Sulforaphane and erucin increase MRP1 and MRP2 in human carcinoma cell lines. *The Journal of Nutritional Biochemistry*.

[10] Cole SPC, Bhardwaj G, Gerlach JH (1992). Overexpression of a transporter gene in a multidrug-resistant human lung cancer cell line. *Science*.

[11] van der Deen M, de Vries EGE, Timens W, Scheper RJ, Timmer-Bosscha H, Postma DS (2005). ATP-binding cassette (ABC) transporters in normal and pathological lung. *Respiratory Research*.

[12] Bréchot J-M, Hurbain I, Fajac A, Daty N, Bernaudin J-F (1998). Different pattern of MRP localization in ciliated and basal cells from human bronchial epithelium. *Journal of Histochemistry and Cytochemistry*.

[13] Scheffer GL, Pijnenborg ACLM, van der Valk P (2002). Multidrug resistance related molecules in human and murine lung. *Journal of Clinical Pathology*.

[14] Manciu L, Chang X-B, Buyse F (2003). Intermediate structural states involved in MRP1-mediated drug transport. Role of glutathione. *The Journal of Biological Chemistry*.

[15] Deeley RG, Cole SPC (2006). Substrate recognition and transport by multidrug resistance protein 1 (ABCC1). *FEBS Letters*.

[16] Thomas GA, Barrand MA, Stewart S, Rabbitts PH, Williams ED, Twentyman PR (1994). Expression of the multidrug resistance-associated protein (MRP) gene in human lung tumours and normal tissue as determined by in situ hybridisation. *European Journal of Cancer A*.

[17] Leier I, Jedlitschky G, Buchholz U, Cole SPC, Deeley RG, Keppler D (1994). The MRP gene encodes an ATP-dependent export pump for leukotriene C4 and structurally related conjugates. *The Journal of Biological Chemistry*.

[18] Karwatsky J, Leimanis M, Cai J, Gros P, Georges E (2005). The leucotriene C4 binding sites in multidrug resistance protein 1 (ABCC1) include the first membrane multiple spanning domain. *Biochemistry*.

[19] Sharom FJ (2008). ABC multidrug transporters: structure, function and role in chemoresistance. *Pharmacogenomics*.

[20] Cao Y, Wang DL, Tao XH, Yang LL, Wang CY, Chen JP (2013). Determination of plasma allylisothiocyanate and its pharmacokinetics in rats by UPLC. *Chinese Journal of Hospital Pharmacy*.

[21] Laupeze B, Amiot L, Courtois A (1999). Use of the anionic dye carboxy-2′,7′-dichlorofluorescein for sensitive flow cytometric detection of multidrug resistance-associated protein activity. *International Journal of Oncology*.

[22] van der Deen M, Homan S, Timmer-Bosscha H (2008). Effect of COPD treatments on MRP1-mediated transport in bronchial epithelial cells. *International Journal of Chronic Obstructive Pulmonary Disease*.

[23] Akan I, Akan S, Akca H, Savas B, Ozben T (2005). Multidrug resistance-associated protein 1 (MRP1) mediated vincristine resistance: effects of N-acetylcysteine and Buthionine sulfoximine. *Cancer Cell International*.

[24] van der Deen M, Marks H, Willemse BWM (2006). Diminished expression of multidrug resistance-associated protein 1 (MRP1) in bronchial epithelium of COPD patients. *Virchows Archiv*.

[25] van der Deen M, Timens W, Timmer-Bosscha H (2007). Reduced inflammatory response in cigarette smoke exposed Mrp1/Mdr1a/1b deficient mice. *Respiratory Research*.

[26] Flens MJ, Zaman GJR, van der Valk P (1996). Tissue distribution of the multidrug resistance protein. *American Journal of Pathology*.

[27] Wright SR, Boag AH, Valdimarsson G (1998). Immunohistochemical detection of multidrug resistance protein in human lung cancer and normal lung. *Clinical Cancer Research*.

[28] Chang QI (2010). Effects of acupoint application on life quality of patients with chronic obstructive pulmonary diseases. *Shanghai Journal of Traditional Chinese Medicine*.

[29] Duan LX (2007). *Chemical constituents of traditional Chinese medicine compound formula Sanziyangqintang and its single medicine the seeds of Raphanus Sativus L. [Ph.D. thesis]*.

[30] Zheng ZW, Zhao Y, Lin QY (1992). The pharmacological studies of Sanziyangqintang. *Pharmacology and Clinics of Chinese Materia Medica*.

[31] Wang LR (2012). The treatment of bronchial asthma of Sanziyangqintang in 120 cases. *Journal of Sichuan of Traditional Chinese Medicine*.

[32] Wang DL, Zhang X, Tao XH (2012). Effects of huatan jiangqi capsule on the levels of multi-drug resistance-associated protein 1 in the bronchial epithelial cells of model rats with chronic obstructive pulmonary disease. *Chinese Journal of Integrated Traditional and Western Medicine*.

[33] Ishii T, Itoh K, Takahashi S (2000). Transcription factor Nrf2 coordinately regulates a group of oxidative stress-inducible genes in macrophages. *The Journal of Biological Chemistry*.

[34] Hayashi A, Suzuki H, Itoh K, Yamamoto M, Sugiyama Y (2003). Transcription factor Nrf2 is required for the constitutive and inducible expression of multidrug resistance-associated protein 1 in mouse embryo fibroblasts. *Biochemical and Biophysical Research Communications*.

[35] Rushworth SA, MacEwan DJ, O’Connell MA (2008). Lipopolysaccharide-induced expression of NAD(P)H:quinone oxidoreductase 1 and heme oxygenase-1 protects against excessive inflammatory responses in human monocytes. *Journal of Immunology*.

[36] He X, Wang L, Szklarz G, Bi Y, Ma Q (2012). Resveratrol inhibits paraquat-induced oxidative stress and fibrogenic response by activating the nuclear factor erythroid 2-related factor 2 pathway. *The Journal of Pharmacology and Experimental Therapeutic*.

